# Enhancing Unconditional Molecule Generation via Online Knowledge Distillation of Scaffolds

**DOI:** 10.3390/molecules30061262

**Published:** 2025-03-12

**Authors:** Huibin Wang, Zehui Wang, Minghua Shi, Zixian Cheng, Ying Qian

**Affiliations:** Shanghai Frontiers Science Center of Molecule Intelligent Syntheses, School of Computer Science and Technology, East China Normal University, Shanghai 200062, China; 51255901010@stu.ecnu.edu.cn (H.W.); 51265901110@stu.ecnu.edu.cn (Z.W.); 51215901061@stu.ecnu.edu.cn (M.S.); 51275901139@stu.ecnu.edu.cn (Z.C.)

**Keywords:** unconditional molecule generation, scaffold-based molecule generation, language model, online knowledge distillation

## Abstract

Generating new drug-like molecules is an essential aspect of drug discovery, and deep learning models significantly accelerate this process. Language models have demonstrated great potential in generating novel and realistic SMILES representations of molecules. Molecular scaffolds, which serve as the key structural foundation, can facilitate language models in discovering chemically feasible and biologically relevant molecules. However, directly using scaffolds as prior inputs can introduce bias, thereby limiting the exploration of novel molecules. To combine the above advantages and address the limitation, we incorporate molecular scaffold information into language models via an **O**nline knowledge distillation framework for the unconditional **M**olecule **G**eneration task (**OMG**), which consists of a GPT model that generates SMILES strings of molecules from scratch and a Transformer model that generate SMILES strings of molecules from scaffolds. The knowledge of scaffolds and complete molecular structures is deeply integrated through the mutual learning of the two models. Experimental results on two well-known molecule generation benchmarks show that the OMG framework enhances both the validity and novelty of the GPT-based unconditional molecule generation model. Furthermore, comprehensive property-specific evaluation results indicate that the generated molecules achieve a favorable balance across multiple chemical properties and biological activity, demonstrating the potential of our method in discovering viable drug candidates.

## 1. Introduction

It has been proposed that there may exist between $10^{23}$ and $10^{60}$ molecules with drug-like properties [1], of which approximately $10^{8}$ have already been synthesized [2]. The cost of developing a drug is substantial, potentially amounting to 1–2 billion USD and a development cycle of 13 years [3,4]. In recent years, advancements in computing technology and artificial intelligence have led to significant breakthroughs in the scientific fields [5]. Deep learning techniques, in particular, have become increasingly competitive with human experts in drug discovery, especially in generating novel molecules, thereby greatly accelerating the drug discovery and design process.

De novo molecule generation, also referred to as unconditional molecule generation, is the fundamental topic of drug discovery and design, as this task provides novel drug candidates with desirable property profiles from scratch [6]. There has been an explosion in the number and variety of generative models that have been applied to molecule generation.

By representing molecules in either topological graphs or the simplified molecular-input line-entry system (SMILES) strings, existing methodologies can be categorized into two primary types based on how the generated molecules are represented: graph-based generation and sequence-based molecular generation.

As molecular structures can be abstracted as graphs in which atoms and bonds are equivalent to nodes and edges, respectively, graph neural networks are intuitive options to represent molecule graphs [7]. Combined with generative models like Variational Autoencoders [8], Normalizing Flows [9], or Diffusion models [10], GNN can generate complete molecules by predicting atom type or bond type iteratively or in parallel. Though the graph-based generation models can find novel and structurally 100% valid molecules, the fixed generation order and constant number of atoms constrain their flexibility.

The emergence of Natural Language Processing techniques offered a novel insight for flexibly generating molecules by treating the SMILES strings analogous to sentences, as the words of sentences can be produced in any order. SMILES strings can be split into atom-level tokens via a regex-based tokenizer [11], and the fundamental concept behind sequence-based molecule generation is to closely approximate the SMILES token distribution that effectively captures the atom-level structure patterns present in a specific dataset. In the early years, generative models [8,12,13] have been proposed to learn such token distributions and good progress has been made. Recently, language generation models like Recurrent Neural Networks (RNNs) [14] and Generative Pre-trained Transformer (GPT) [15] have excelled at understanding and generating realistic natural language, making them crucial for tasks that demand deep textual creation [16]. In the field of drug discovery and design, they have demonstrated great potential in generating novel and realistic SMILES strings [17].

Every molecule has a core substructure known as a scaffold, which consists of a set of connected atoms that form a structural base for the molecule, excluding the side chains and functional groups. The scaffolds define a molecule’s basic shape, size, and stereochemical properties, significantly influencing its physicochemical and biological characteristics, making them a key consideration in molecular design [18]. Despite the enhancement in discovering chemically feasible and biologically relevant molecules, most scaffold-based methods rely on predefined patterns and expert knowledge. This imposes a limitation on querying arbitrary kinds of scaffolds when generating molecules. To address this limitation, some approaches adopt a two-step generation process: they first generate a new scaffold and then complete the molecular structure based on that scaffold [19,20]. However, explicitly providing prior molecular scaffolds will lead to bias of observed structures and still limit the search space of molecules with novel structures.

To combine the strengths of the language generative models and the scaffold-based molecular generation methods and solve the above limitations, we proposed an **O**nline knowledge distillation framework for learning unconditional **M**olecule **G**eneration (OMG). The OMG architecture comprises two models: a GPT model that generates a SMILES string of a complete molecule from scratch (OMG-GPT) and a Transformer model that generates the SMILES string of a molecule from its scaffold (OMG-SG). The above two models mutually learn to generate the same molecules by aligning their feature maps and logits, respectively. This mutual learning strategy enables the integration of scaffold information into the GPT model, thus enhancing the language model’s awareness of key molecular structures while preserving its capacity to generate novel molecules.

We conducted comprehensive evaluation experiments for the models trained in the distillation framework on the public MOSES [21] and GuacaMol [22] datasets for molecule generation. The experimental results demonstrate that, compared to most existing state-of-the-art (SOTA) methodologies, OMG-GPT achieves optimal performance across validity and novelty. Furthermore, OMG-GPT generated molecules containing novel scaffolds and molecules not observed in the existing molecule databases, and the generated molecules exhibit rational properties as they adhere to the chemical property and biological activity distributions of the original datasets. The above findings also demonstrate the potential of our method in structurally novel and pharmaceutically favorable drug candidates.

## 2. Methods

**Figure 1:**
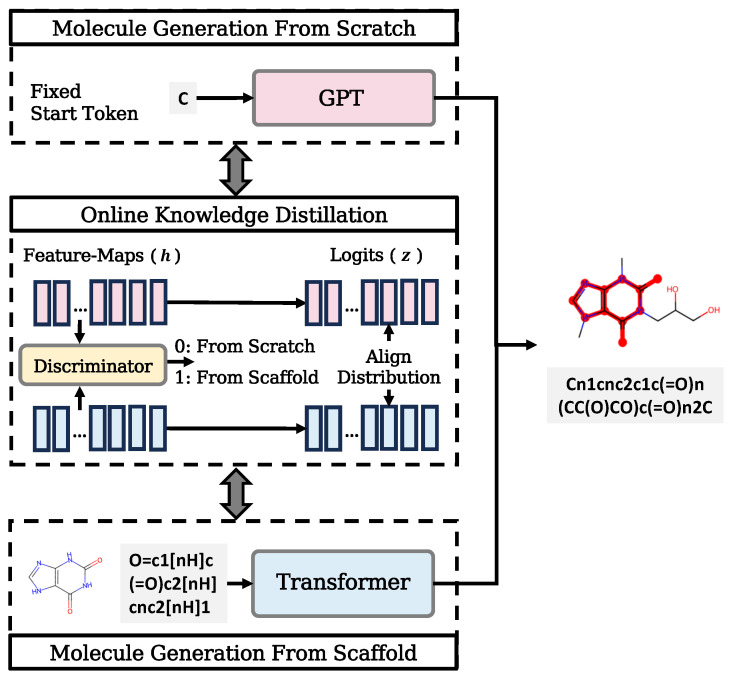
Illustration of our proposed **O**nline knowledge distillation for **M**olecule **G**eneration (OMG). A GPT model that generates a SMILES string of a complete molecule from the fixed start token ‘C’ (OMG-GPT) and a Transformer model that generates a SMILES string of the same molecule from its scaffold (OMG-SG) are closely coupled through online knowledge distillation in both feature map level and logit level.

We introduce OMG, the online knowledge distillation framework that employs mutual learning at the logit level and adversarial distillation learning at the feature map level to integrate scaffold information into the unconditional molecule generation task. This approach enables the model that generates molecules from scratch to learn from a scaffold-based molecule generation model; the involved models are both implemented based on language models. The method schematic is illustrated in Figure 1.

### 2.1. Online Knowledge Distillation Framework

The OMG training framework includes two models: OMG-GPT, which performs the unconditional molecule generation task, and OMG-SG, which conducts the scaffold-based molecule generation task, respectively. The OMG-GPT model uses a Decoder-Only GPT architecture to generate the SMILES representations of molecules from scratch, following the next-token prediction paradigm. The OMG-SG model includes an Encoder-Decoder Transformer architecture, which incorporates SMILES of scaffolds and generates complete molecules. The final objective of the training framework is to align the SMILES token distribution from OMG-GPT and OMG-SG with the SMILES token distribution of the reference molecules in the dataset. This framework enables OMG-GPT to generate molecules based on aligned implicit prior scaffold information, avoiding the problem of model bias.

The online distillation process consists of two methods: mutual learning and adversarial distillation. Mutual learning is implemented by minimizing the KL divergence between the logits of SMILES tokens to be selected. Adversarial distillation is implemented by introducing an LSTM-based discriminator to align the dynamic molecular features output from the two models. Two Cross-Entropy losses lead to the two models producing reference molecules.

### 2.2. Mutual Learning

Deep Mutual Learning (DML) [23] is used to train two identical neural networks based on the same data, which belongs to the online knowledge distillation family. It is suggested that even if the training objectives of the two networks are the same, differences in the initial conditions and the generative approaches provide different information from both sides during training. Despite the internal differences, the final logit output from the networks should agree with the same ground truth, which forces the two networks to eventually behave consistently.

Unlike conventional DML, two models with different architectures are involved in the training framework. Although both models aim to generate complete molecules from the dataset, their focuses differ. The OMG-GPT focuses purely on fitting the token distribution in the dataset, while the OMG-SG model concentrates on fitting the token distribution based on the prior conditions of input molecule scaffolds. To enable the OMG-GPT to learn the structural information of the scaffolds, we use KL divergence to tighten the logit distributions of the generated tokens from the two models. Differently from DML, we only calculate the divergence from the distribution of OMG-GPT to that of OMG-SG, encouraging the former to learn more scaffold knowledge. Here, the KL divergence is defined as follows, where $P(z_{1})$ represents the distribution of logit output from the OMG-GPT, and $P(z_{2})$ represents the distribution of output from the OMG-SG:

(1) $$L_{mutual}=D_{KL}(P\left(z_{1}\right)||P\left(z_{2}\right))=\int P\left(z_{1}\right)\log\frac{P(z_{1})}{P(z_{2})}dz_{1}.$$

Additionally, we updated the parameters of both models simultaneously, rather than alternately updating the parameters for each one, which increases training efficiency.

In summary, mutual learning aligns the logit-level distribution of OMG-GPT and that of OMG-SG towards the same target SMILES token distributions. This process initially enables the integration of scaffold information into OMG-GPT.

### 2.3. Adversarial Distillation

Feature maps are crucial intermediate representations during the mutual learning-based online distillation process, capturing meaningful molecular structure information. Unlike offline methods, online distillation does not have a static target to follow at the feature map level [24]. Moreover, compared to logits constrained by the class probability of final tokens, feature maps undergo more flexible and drastic changes during training. Directly minimizing the distance between the two feature map points via the $L_{1}$ or $L_{2}$ norm ignores the distributional difference, and the KL divergence is highly sensitive to such differences.

To address this issue, we adopted the concept of Generative Adversarial Networks (GANs) as proposed in [24] to align the feature-map distributions effectively. Specifically, a discriminator is employed to differentiate whether the generated features originate from the OMG-GPT or the OMG-SG.

In the training framework, the output from the last layer of the decoder belonging to each model is selected as the feature map of a molecule. The discriminator is implemented by a two-layer bidirectional LSTM (Bi-LSTM) neural network [25] that incorporates the feature map and yields a scaler between 0 and 1, which represents whether the features are generated based on a scaffold. The loss for the unconditional molecule generator is expressed as:

(2) $$L_{gen}=log\left(1-D\left(h_{1}\right)\right).$$

where term $h_{1}$ represents the feature maps output from the OMG-GPT, and the term D(*) represents the output of the discriminator. The loss for the discriminator is expressed as:

(3) $$L_{d}=\frac{log(D\left(h_{2}\right))+log(1-D\left(h_{1}\right))}{2},$$

where $h_{2}$ represents the feature maps of OMG-SG.

The feature maps $h_{1}$ (or $h_{2}$) are projected to logit $z_{1}$ (or $z_{2}$) to ensure the coherence of the data flow and enable end-to-end training. The end-to-end training strategy enables the molecular features generated from scratch to better align with those derived from scaffolds, thereby further integrating structural information.

### 2.4. Training

The total loss for the generation part of OMG is as follows:

(4) $$Loss=L_{gen}+\lambda_{1}L_{CE}^{1}+\lambda_{2}L_{CE}^{2}+\lambda_{3}L_{mutual},$$

where $L_{CE}^{1}$ ($L_{CE}^{2}$) [26] represent the Cross-Entropy loss that calculates difference between the predicted SMILES tokens based on the logits of OMG-GPT (OMG-SG) and the ground truth token of the target molecule, respectively. The term $L_{mutual}$ represents the loss of the mutual learning mechanism, as described in Equation (1). The coefficients $\lambda_{1}$, $\lambda_{2}$, $\lambda_{3}$ are the weights assigned to each loss.

Optimization of the loss $L_{d}$ in Equation (3) is separated from the total loss. To prevent the discriminator from becoming overly dominant and hindering OMG-GPT’s ability to effectively mimic the generation process based on scaffolds, $L_{d}$ is computed and updated once for each five training steps.

## 3. Experiments Configuration

### 3.1. Datasets

In this work, we used two public benchmark datasets, MOSES [21] and GuacaMol [22], to evaluate the performance for the unconditional molecule generation task. MOSES is a dataset containing 1.9 million lead-like molecules extracted from the ZINC database [27]. These molecules exhibit specific properties: their molecular weight ranges between 250 and 350 Da, they have fewer than 8 rotatable bonds, and their XlogP value is less than 3.5. Additionally, they possess good biological activity, making them excellent starting points for potential drug candidate molecules. MOSES contains a scaffold split test set (TestSF). All molecules in the TestSF set have molecular scaffolds that are not present in the training set. GuacaMol is a subset of ChEMBL [28], comprising 1.6 million molecules. The Bemis-Murcko scaffolds of these molecules are computed using RDKit (2023.9.6) [29].

**Figure 2:**
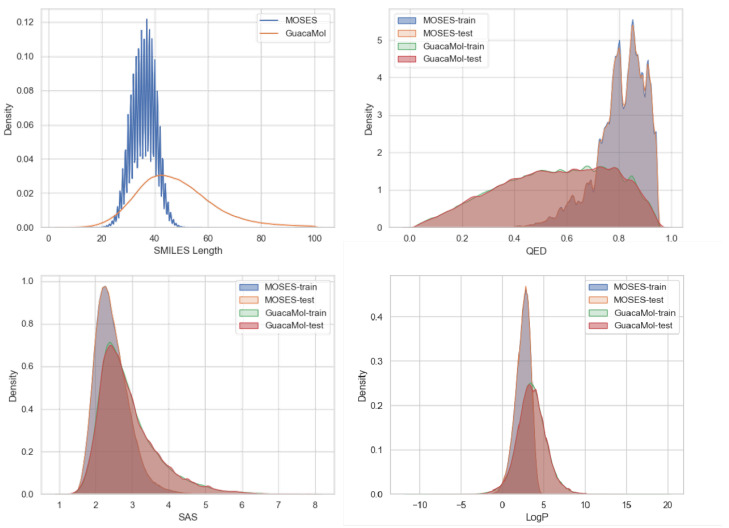
Probability distributions of properties, including SMILES Length, QED (Quantitative Estimate of Drug-likeness), SAS (Synthetic Accessibility Score), and LogP (Octanol–Water Partition Coefficient) of molecules in the MOSES and GuacaMol datasets.

Figure 2 illustrates the differences in the probability distributions of chemical properties between the two datasets, in which the property values were also calculated using RDKit (2023.9.6). The molecules in GuacaMol have longer average SMILES lengths compared to those in MOSES, with a more pronounced difference in SMILES length. Additionally, the distributions of chemical property values are more dispersed and uniform in GuacaMol. These characteristics indicate that the GuacaMol dataset presents a greater level of complexity for molecule generation tasks compared to MOSES. The distributions of the properties in the training set and the test set in both datasets are basically the same, which is also considered essential for ensuring unbiased evaluation and consistent generalization of generative models during the training and testing phases.

### 3.2. Evaluation Metrics

The metrics used to evaluate the performance of unconditional molecule generation are as follows:

- **Validity**: Validity refers to the proportion of valid molecules among those generated. A molecule is considered valid if it conforms to the rules of molecular structure, such as proper valence and absence of forbidden structures.
- **Uniqueness**: Uniqueness refers to the proportion of unique molecules among the valid molecules generated. High uniqueness indicates that the model frequently generates distinct molecules, suggesting that it has learned the variance in the distribution well [21].
- **Novelty**: Novelty refers to the proportion of valid molecules generated that do not appear in the training set. This metric evaluates the model’s ability to generate new molecules that are potentially useful but have not been explored previously.
- **Internal Diversity (${\mathrm{IntDiv}}_{p}$)**: This metric assesses the diversity among the molecules generated. It is important to determine whether the model has generated a variety of distinct molecular structures or just similar molecules. Internal diversity is typically calculated using a pairwise similarity measure, most commonly the Tanimoto similarity [30] based on molecular fingerprints. The corresponding formula is as follows:
  (5) $$IntDiv_{p}\left(S\right)=1-\sqrt[{p}]{\frac{1}{{\left|S\right|}^{2}}T{\left(s_{1},s_{2}\right)}^{p}},$$
  where the terms $s_{1}$ and $s_{2}$ represent the molecules in the generated set *S*. The term $IntDiv_{p}$ represents the power (*p*) mean of Tanimoto similarity (*T*). In this work, we report $IntDiv_{1}$ and $IntDiv_{2}$.
- **Scaffold Similarity (Scaff)**: This metric compares the distribution of Bemis–Murcko scaffolds [31] of molecules in the reference and generated sets. Denoting $c_{s}\left(A\right)$ as the number of times a given scaffold *s* appears in a set of molecules *A*, and a set of scaffolds that appear in either the generated set *G* or the reference set *R* as *S*, the metric is defined as a cosine similarity:
  (6) $$\mathrm{Scaff}\left(G,R\right)=\frac{\sum_{s\in S}\left(c_{s}\left(G\right)\cdot c_{s}\left(R\right)\right)}{\sqrt{\sum_{s\in S}c_{s}^{2}\left(G\right)}\sqrt{\sum_{s\in S}c_{s}^{2}\left(R\right)}}.$$
  The purpose of this metric is to show how similar the scaffolds are that are present in the generated and reference datasets [21]. In this paper, we report Scaff/TestSF, where the scaffolds in the test set are selected as the reference set. The score of Scaff/TestSF reveals the model’s capability to discover novel scaffolds.

A good generative model should try to generate a greater number of novel, valid molecules so as to help us explore that chemical space, high values of novelty, uniqueness, and validity would ensure that the models have learned the molecule grammar well and are not overfitting to the training data simultaneously. Internal diversity quantifies the extent of chemical space traversed by the generative model. Scaffold similarity measures the generative model’s ability to integrate the scaffold information of reference molecules.

In drug design, it is essential for a new compound with a novel scaffold to exhibit desirable chemical and biological properties. Therefore, generative models must be capable of effectively producing molecules that demonstrate strong chemical property and biological activity scores. Several metrics are used to evaluate these properties:

- **Quantitative Estimation of Drug-likeness (QED)**: QED is a value in the range of [0, 1] that estimates the likelihood of a molecule being a viable candidate for a drug. A higher QED value indicates a greater likelihood of the molecule having drug-like properties.
- **Synthetic Accessibility Score (SAS)**: The Synthetic Accessibility Score (SAS) is a heuristic measure that estimates the difficulty of synthesizing a given molecule. The score ranges from 1 (easy to synthesize) to 10 (hard to synthesize).
- **LogP**: The logarithm of the partition coefficient (LogP) measures the solubility of a solute between two immiscible solvents at equilibrium. This value is calculated using RDKit’s Crippen [32] estimation method. The ideal LogP value for a drug typically falls within the range of 1 to 5.
- **pIC50**: The IC50 value represents the molar concentration of an inhibitor (such as a drug) needed to inhibit 50% of a biological function or target, such as an enzyme, cell receptor, or microbe. pIC50 represents the negative logarithm of the IC50, and the high pIC50 value indicates that the tested molecule possesses better biological activity.

### 3.3. Implementation Settings

The models were implemented based on the deep-learning library Pytorch (version: 2.3.0) [33]. The unconditional generative model OMG-GPT employs a GPT architecture with eight decoder layers, while the scaffold-based model OMG-SG utilizes a standard Transformer architecture that includes eight encoder layers and eight decoder layers. Both models feature an eight-head attention mechanism, and the dimensionality of the features is set to 256. We selected the AdamW optimizer for optimization, with beta parameters set to (0.9, 0.98) and an initial learning rate of 0.0006. We used another AdamW optimizer for the discriminator, changing the initial learning rate to 0.0003. A Pytorch GradScaler [33] was used to adjust the learning rate during the training process. Three loss weights $\lambda_{1}$, $\lambda_{2}$, $\lambda_{3}$ were treated as hyper-parameters optimized through experiments (refer to Section S1 in the Supplementary Material). Based on these experiments, the weights were set to 1.5, 1, and 0.5 for both datasets. The batch size for training on the MOSES dataset was 512, while on the GuacaMol dataset, it was 300. For both datasets, the two models were trained on an NVIDIA GeForce RTX 4090 GPU (Santa Clara, CA, USA) in 100 epochs with a time cost of approximately 12 h.

## 4. Results and Discussion

In the unconditional molecule generation task, an effective generative model should produce a higher proportion of valid and novel molecules. To assess this, OMG-GPT was trained and evaluated on the MOSES and GuacaMol datasets using the evaluation metrics outlined in Section 3.2. Except for validity, all other metrics were computed based on the subset of valid molecules generated by the model.

We also compared the OMG-GPT with several existing molecular generation methods, which are categorized into two approaches: graph-based and sequence-based methods. GraphINVENT [34], JT-VAE [35], NAGVAE [36], and Digress [10] are recent graph-based methods. GraphINVENT generates a molecule graph by iteratively predicting a node and a bond. JT-VAE generates a molecular graph in two phases by first generating a tree-structured scaffold over chemical substructures and then combining them into a molecule. NAGVAE compresses the generation process of the molecular graph via decomposing the graph into several substructural patterns. Digress utilizes a discrete diffusion process to generate the molecular graph in one shot [10]. Digress reports results on both MOSES and GuacaMol, while JT-VAE and GraphINVENT only report results on the MOSES dataset, and NAGVAE only reports results on the GuacaMol dataset. CharRNN [37], VAE [8], LatentGAN [38], SMILES LSTM [37], ORGAN [13], Sc2Mol [20], and MolGPT [17] are representative sequence-based methods that generate molecular SMILES strings using the next-token prediction paradigm. The exception is Sc2Mol, which uses a two-step generation process. In Sc2Mol, the first step generates the SMILES of a scaffold, and the second step generates the final molecule based on the scaffold. Among them, AAE, VAE, and MolGPT report results on both datasets. The remaining models only report results on the MOSES dataset. The comparison was performed across 10,000 molecules generated by each model.

### 4.1. Performance Comparison on the MOSES Datasets

The results on the MOSES dataset are shown in Table 1. Except for JT-VAE, which consistently generates valid molecules at every step of generation, OMG-GPT outperformed other methods in terms of validity, achieving 0.99. This high validity demonstrates that OMG-GPT effectively captures the distribution of valid SMILES tokens. Additionally, OMG-GPT achieved 100% in unique rate and delivered a competitive performance in novelty. Specifically, compared to other models with validity > 90%, OMG-GPT showed a significant advantage in novelty, with a margin exceeding 16%. Conversely, models that surpass OMG-GPT in novelty achieved validity scores below 90%, highlighting a clear gap between them and our model. It is important to note that both validity and novelty are crucial for the generated molecules, and our results demonstrate a good balance between the two. Regarding Scaff/TestSF, which is associated with the core substructures, OMG-GPT achieved the highest score, suggesting that the scaffolds generated by our model are closer to those observed in the dataset. These findings further demonstrate that the OMG framework effectively learns scaffold information, laying a solid foundation for generating valid molecules. The improvements in all metrics compared to MolGPT indicate that our proposed framework effectively mitigates the overfitting issue in the pure GPT model, thereby enhancing the GPT model’s capability to generalize to unexplored chemical spaces.

### 4.2. Performance Comparison on the Guacamol Datasets

The results on the GuacaMol dataset, presented in Table 2, show that OMG-GPT achieved the highest validity while maintaining strong novelty across both graph-based and sequence-based methods. These results underscore again that OMG-GPT effectively balances validity and novelty, ensuring that the generated molecules are both chemically sound and structurally diverse.

Compared to its performance on the MOSES dataset, OMG-GPT showed a slight 1% decrease in validity but exhibited a notable 7% improvement in novelty. This trend suggests that with longer generated SMILES sequences, OMG-GPT gains greater potential to explore novel molecular structures while maintaining high structural validity. These findings further highlight the robustness of the OMG framework in capturing meaningful molecular structure distributions and enhancing generalization in unexplored chemical spaces, thus providing more new samples for the study of chemical molecular structures.

### 4.3. Ablation Experiments

We conducted additional experiments on the MOSES dataset to investigate the effects of our proposed methods on unconditional molecular generation in terms of individual knowledge distillation modules and different knowledge distillation approaches.

#### 4.3.1. Effects of Individual Knowledge Distillation Modules

To further investigate the effects of our proposed modules, we conducted ablation experiments using variants of the OMG-GPT. We removed the mutual learning module, which is represented as w/o ML. The model with the removal of the adversarial distillation module is represented as w/o Ad. By removing all the proposed modules, we retained the original MolGPT. To investigate the benefits of online knowledge distillation itself, we replaced the Transformer that performs scaffold-based generation with another GPT model that generates molecules from scratch with different parameter initialization, creating Dual-GPT. The results of the ablation experiment are shown in Table 3.

Mutual learning is the most critical part, as it significantly improved the novelty by 7.4% for the GPT model. Adversarial distillation at the feature map level further enhances the novelty combined with mutual learning, which is supported by the improvement when comparing OMG and OMG w/o Ad. Only using the adversarial distillation at the feature map level during training degraded the generation performance of the GPT model, resulting in a 5.5% reduction in novelty when comparing OMG w/o ML to MolGPT. The reduction shows that when mutual learning at the logit level is not used, the feature maps of molecules from the GPT face a challenge in aligning with those produced based on scaffolds. The above results also indicate that alignment at the logit level, as a further target of online distillation, can tighten alignment at the feature map level and facilitate the transfer of molecular scaffold knowledge.

In order to verify the effectiveness of introducing the discriminator, we replaced the process of adversarial distillation with minimizing the KL divergence between the two feature maps during training, resulting in OMG-GPT (MF). The results in Table 3 show that the performance of OMG-GPT (MF) is close to, yet still inferior to, the performance of OMG w/o Ad. This outcome highlights that simple KL divergence minimization is insufficient to effectively align the feature map distributions, further underscoring the distinct differences between aligning at the logit level and aligning at the feature map level.

Dual-GPT showed 3.2% lower validity compared to MolGPT but achieved 14% higher novelty, even surpassing OMG-GPT. This indicates that the online distillation itself can enhance the exploration of diverse outcomes, though it comes with the drawback of reduced learning of valid SMILES structures.

#### 4.3.2. Effects of Different Knowledge Distillation Approaches

To further investigate the difference in effects brought by different knowledge distillation approaches on learning scaffold information, we applied a traditional knowledge distillation framework for comparison. In this setup, a pre-trained Transformer for scaffold-based generation was used to guide the pure GPT model, creating KD-GPT. Additionally, we compared the scaffold-based generation models trained in the two frameworks: OMG-SG, which was trained in the proposed online knowledge distillation framework, and KD-SG, which was trained in the traditional knowledge distillation framework. For evaluation, we also sampled 10,000 molecules generated from each GPT-based model and sampled 1000 molecules generated from each scaffold-based generation model with the same input scaffold string ‘(O=C(COc1ccccc1)Nc1ccccc1)’. The results are presented in Table 4.

Among the GPT-based models, although KD-GPT showed no reduction in validity compared to OMG-GPT, its novelty decreased and even fell below that of MolGPT. This indicates that traditional distillation, relying solely on previously learned scaffold knowledge, limits the exploration of diverse molecular structures.

The novelty of molecules generated by OMG-SG was significantly higher than that of KD-SG, albeit it exhibited a slight disadvantage in validity. This suggests that knowledge from the OMG-GPT contributes to enhancing OMG-SG’s performance in scaffold-based molecule generation within the online distillation framework.

These findings demonstrate the effectiveness of the proposed online distillation framework, which enables bidirectional knowledge transfer between models. Unlike traditional one-way distillation, this approach integrates complementary knowledge from both scaffold-based and de novo generation, enhancing scaffold learning and promoting molecular diversity for improved performance.

### 4.4. Property-Specific Evaluations of the Generated Molecules

Building on the validation of the OMG framework’s effectiveness in facilitating the discovery of novel and valid molecules, we further evaluated its ability to promote the discovery of molecules with desired chemical and biological properties.

#### 4.4.1. Chemical Properties Distribution

**Figure 3:**
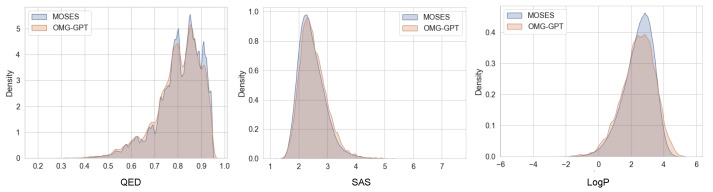
Comparison of the QED (Quantitative Estimate of Drug-likeness), SAS (Synthetic Accessibility Score), and LogP (Octanol–Water Partition Coefficient) distributions for molecules in the MOSES train set and those generated by OMG-GPT (10,000 molecules).

**Figure 4:**
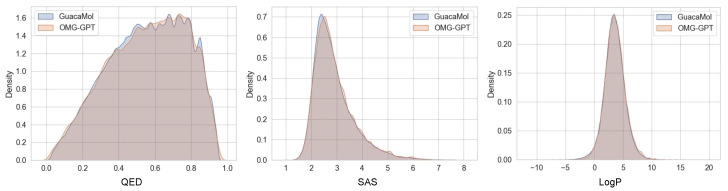
Comparison of the QED (Quantitative Estimate of Drug-likeness), SAS (Synthetic Accessibility Score), and LogP (Octanol–Water Partition Coefficient) distributions for molecules in the GuacaMol train set and those generated by OMG-GPT (10,000 molecules).

We assess the chemical properties (QED, SAS, LogP) distribution fitting performance of OMG-GPT on the MOSES and GuacaMol datasets. Specifically, we first calculated the values of 10,000 generated molecules and the original molecules for each property, then plotted kernel density estimation curves. Finally, we compared the proximity of the property distribution curves of the two sets of molecules to assess the OMG-GPT’s performance in learning the chemical property distribution. The comparison of the curves on the MOSES and Guacamol datasets is shown in Figure 3 and Figure 4.

Overall, OMG-GPT effectively aligned the property distributions of the two original datasets. Additionally, compared to the distribution of the original datasets, OMG-GPT exhibited a lower probability density for intermediate property values and higher density at the extremes. These results indicate that OMG-GPT generates molecules with more pronounced property variations, demonstrating the capability of the OMG framework to capture the diverse and complex relationships between SMILES strings and their corresponding properties.

#### 4.4.2. Biological Activity Distribution

To assess the biological activity of the molecules generated by our unconditional generation model, we adopted the experimental framework from [39], focusing on five human protein targets: CDK2, EGFR, JAK1, LRRK2, and PIM1. Drug–target affinity predictions were performed using GraphDTA [40], a deep learning-based method that takes in the protein sequence and molecular graph and predicts the pIC50 value. Furthermore, we included molecules from the MOSES and Guacamol datasets in our experiments with the same five protein targets to serve as a reference. The pIC50 values’ mean and variance were computed to represent the overall distribution of biological activity against these proteins. The results of the experiments are provided in Table 5.

Overall, the distribution of pIC50 scores for the molecules generated by the OMG-GPT model closely aligns with that of the molecules in the original dataset. This indicates that the molecules generated by our model exhibit biological activity patterns consistent with the activity distributions observed in existing databases. Specifically, although the molecules generated by the model have a lower average pIC50 for the EGFR protein than those in the original dataset, the average activity of the generated molecules is higher for the remaining proteins. In particular, compared to the molecules in the MOSES dataset, the mean values of pIC50 for the generated molecules increased by 1.15% on average. This suggests that our model, while remaining in line with established activity profiles, is capable of identifying molecules with better activity.

### 4.5. Case Study

Based on the satisfied generation results, we further evaluate the model’s performance in discovering truly novel scaffolds and molecules. Specifically, we count the molecules whose scaffolds are not observed in the two datasets and molecules that are not present in the PubChem database [2] among the 10,000 molecule samples generated by OMG-GPT. For the MOSES dataset, OMG-GPT generated 26 molecules with scaffolds not present in the dataset, 16 of which were not found in the PubChem database. For the GuacaMol dataset, OMG-GPT generated 4941 molecules with scaffolds not present in the dataset, and 4779 of these molecules were not found in the PubChem database. This indicates that the OMG-GPT model is capable of generating a significant number of novel molecular structures, both in terms of scaffolds and individual molecules.

**Figure 5:**
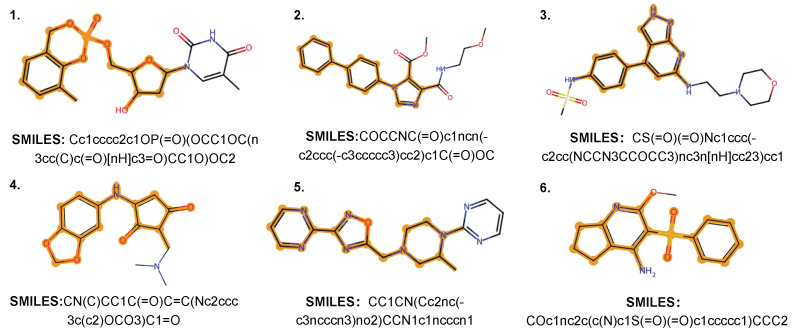
Examples of molecules with novel scaffolds generated by OMG-GPT that are not found in existing datasets or the PubChem [2] database. The generated SMILES strings are converted into molecular graphs using RDKit [29]. The molecular scaffolds are extracted using ScaffoldGraph [42] and are highlighted in orange.

Furthermore, to systematically examine the utility of the molecules generated with novel scaffolds that do not appear in the current databases, we selected QED, SAS, and the pIC50 values for five proteins as a set of objectives. By solving the Pareto front set [41], we identified 627 molecules that performed the best across multiple properties from the molecule set generated by the OMG-GPT trained on the Guacamol dataset. Furthermore, with the constraints of LogP values in the range [1, 3] and QED > 0.5, we selected six molecules with the highest activity on specific proteins or the highest QED values. The resulting molecular structures are shown in Figure 5, and the corresponding property values for each molecule are displayed in Table 6. The molecules in Table 6 that achieve the best values in one property metric also perform well across other property metrics. This suggests that our method holds significant potential for discovering novel drug molecules that meet a wide range of practical requirements, and it may even contribute to expanding existing pharmaceutical molecule databases.

### 4.6. Limitations

Despite the promising performance of OMG in producing novel and potentially useful drug molecules, there are still three limitations that need to be addressed to facilitate its widespread application in drug discovery and design.

The first limitation is that our method relies on scaffold information in a 1D SMILES format, which does not fully capture the spatial structures of the scaffolds and the three-dimensional (3D) space. Given that 3D molecular structure information is more crucial for understanding protein–ligand interactions, enzymatic functions, and a range of other biomolecular phenomena [43], this limitation restricts our model’s ability to discover new molecules that meet more specific medical requirements. One possible solution is to project the atomic coordinates into the input SMILES token space [44,45].

Another limitation is that our method lacks explicit learning of the relationship between molecular structures and their functional context, making it difficult to effectively respond to demands when generating molecules with specified functions like better drug-likeness or higher binding-affinity score. A promising solution is to introduce multi-task learning related to the properties of the generated molecules, adding additional loss functions, such as those predicting the corresponding property values [46] or property rankings [47] to the standard loss function for reconstructing SMILES strings.

Based on the limitations mentioned above and potential solutions, we also found that the evaluation metrics we currently use do not comprehensively cover the expanded task context. For example, scaffold similarity metrics only analyze structural similarity and do not account for biological relevance. Finding novel structures while ensuring molecules meet specific property targets is also crucial, especially in scaffold hopping and lead optimization [48]. To address this issue, we propose incorporating a substructure-constrained evaluation approach, which involves calculating the success rate of generating molecules that achieve specific properties, such as LogP or binding energy within a 3D protein pocket, while maintaining a defined structural similarity to the reference molecules [46].

## 5. Conclusions

In this study, we present the **OMG** training framework for sequence-based unconditional molecule generation tasks. The framework allows a pure GPT model (OMG-GPT) that generates molecules from scratch and a scaffold-based generation model (OMG-SG) to learn from each other, integrating scaffold information into the GPT model while maintaining its ability to generate novel molecules. OMG-GPT demonstrates competitive performance across various benchmark metrics, outperforming most methods in generating valid and novel molecules. It also achieves the highest scaffold similarity compared to molecules in the Guacamol dataset. These findings emphasize that scaffold information is effectively integrated into the molecule generation models within our OMG framework. Further, OMG-GPT generates molecules not presented in the existing databases, with their scaffolds not observed in the original datasets. These molecules also exhibit chemical property values and biological activity scores that align with the overall distributions of properties and activities observed in the original datasets, demonstrating that our method can identify potentially viable drug candidates.

In summary, our proposed OMG framework provides valuable insights into integrating key structural information to enhance molecule generation. Future efforts will focus on incorporating 3D structural information of molecules and introducing more learning and evaluation tasks that account for both structural constraints and property-related objectives. Additionally, we aim to integrate large language model techniques to ultimately enable the flexible generation of more practical molecules, tailored to specific targets.

## Figures and Tables

**Table 1:** Comparison of the performances of different approaches for unconditional molecule generation across 10,000 samples on the MOSES dataset.

Models	Validity	Unique	Novelty	${\mathrm{IntDiv}}_{1}$	${\mathrm{IntDiv}}_{2}$	Scaff/TestSF
**Graph-Based**						
JT-VAE [35]	**1.0**	0.999	0.914	0.855	0.849	0.101
GraphINVENT [34]	0.964	0.998	-	0.857	0.851	0.127
Digress [10]	0.857	1.0	0.936	0.855	0.849	0.118
**Sequence-Based**						
CharRNN [37]	0.975	0.999	0.842	0.856	0.850	0.110
AAE [12]	0.936	**1.0**	0.793	0.855	0.850	0.079
VAE [8]	0.977	0.998	0.700	0.856	0.850	0.058
LatentGAN [38]	0.897	0.997	0.949	0.857	0.850	0.107
MolGPT [17]	0.988	**1.0**	0.821	0.855	0.849	0.085
Sc2Mol [20]	0.631	0.990	**0.986**	**0.866**	**0.872**	0.081
OMG-GPT (ours)	0.990	**1.0**	0.903	0.855	0.849	**0.134**

The results are obtained through reproduced experiments. The best performance is in **bold**, and the second-best performance is underlined.

**Table 2:** Comparison of the performances of different approaches for unconditional molecule generation across 10,000 samples on the GuacaMol dataset.

Models	Validity	Unique	Novelty
**Graph-Based**			
Digress [10]	0.852	**1.0**	0.996
NAGVAE [36]	0.929	0.955	**1.0**
**Sequence-Based**			
SMILES LSTM [37]	0.959	**1.0**	0.912
VAE [8]	0.870	0.999	0.974
AAE [12]	0.822	**1.0**	0.998
ORGAN [13]	0.379	0.841	0.687
MolGPT [17]	0.979	0.999	**1.0**
OMG-GPT (ours)	**0.984**	**1.0**	0.975

The results of MolGPT are obtained through reproduced experiments. The remaining results are from the published papers. The best performance is in **bold**, and the second-best performance is underlined.

**Table 3:** Comparison of the performance of OMG variants for unconditional molecule generation on the MOSES dataset. OMG-GPT (MF) denotes the GPT model trained in our proposed online knowledge distillation framework, where the adversarial distillation is replaced with the calculation of KL divergence between the feature maps. Dual-GPT denotes the GPT model trained in our proposed online knowledge distillation framework in which the scaffold-based generation model is replaced by another GPT model.

Models	Validity	Unique	Novelty
OMG	**0.990**	**1.0**	0.903
OMG w/o ML	0.988	**1.0**	0.766
OMG w/o Ad	**0.990**	**1.0**	0.895
MolGPT	0.988	**1.0**	0.821
OMG-GPT (MF)	0.983	**1.0**	0.893
Dual-GPT	0.956	**1.0**	**0.961**

The best performance is in **bold**.

**Table 4:** Performances on molecule generation from scratch (the upper group of rows) and scaffold-based molecule generation (the lower group of rows) on the MOSES dataset. OMG-GPT and OMG-SG, respectively, denote the generation models trained in our proposed Online Knowledge Distillation framework. KD-GPT and KD-SG denote the generation models trained in the traditional Knowledge Distillation framework. All the results were obtained through reproduced experiments.

Models	Validity	Unique	Novelty
OMG-GPT	**0.990**	**1.0**	**0.903**
MolGPT	0.988	**1.0**	0.821
KD-GPT	**0.990**	**1.0**	0.776
OMG-SG	0.978	**1.0**	**0.917**
KD-SG	**0.996**	**1.0**	0.772

The best performance in each group row is in **bold**.

**Table 5:** Statistics and comparison of biological activity for the molecules generated by the OMG-GPT and that of the original datasets.

Source of Molecules	pIC50 (Mean/Std)				
	CDK2	EGFR	JAK1	IRRK2	PIM1
MOSES	5.35/0.62	5.39/0.28	5.22/0.26	5.67/0.19	5.75/0.34
${\text{OMG-GPT}}_{M}*$	5.46/0.59	5.37/0.29	5.32/0.29	5.76/0.19	5.91/0.38
Guacamol	5.65/0.60	5.62/0.40	5.34/0.49	5.88/0.28	6.18/0.64
${\text{OMG-GPT}}_{G}*$	5.67/0.64	5.60/0.39	5.34/0.49	5.89/0.28	6.23/0.65

The subscript _M_* means the GPT model was trained on the MOSES dataset and the subscript _G_* means training on the Guacamol dataset.

**Table 6:** Statistics of values of multiple properties for each molecule enumerated in Figure 5. Each row of record corresponds to a molecule in the figure based on the number ID.

ID Number	LogP	QED ↑	SAS ↓	pIC50 ↑				
				CDK2	EGFR	JAK1	IRRK2	PIM1
1	1.54	0.71	4.21	**6.64**	5.13	4.80	5.14	5.27
2	2.70	0.50	**2.24**	5.07	**6.49**	5.29	5.16	5.18
3	1.74	0.54	2.76	6.00	4.78	**6.62**	4.80	6.77
4	1.04	0.84	3.29	5.48	4.98	4.99	**6.98**	5.53
5	1.03	0.69	3.04	5.70	4.16	4.87	5.43	**7.33**
6	1.99	**0.94**	2.46	5.08	5.45	4.72	5.31	5.26

The upper arrow mark indicates that higher values are better, while the down arrow indicates the opposite. The best value for each metric is in **bold**.

## Data Availability

All code of the proposed method and datasets used are open-sourced in the GitHub repository: https://github.com/xxxlive/AMLGPT, (accessed on 8 March 2025).

## Supplementary Materials

The following supporting information can be downloaded at: https://www.mdpi.com/article/10.3390/molecules30061262/s1, Table S1: Comparison of OMG’s performance with different weights of losses on the MOSES dataset; Table S2: Experimental results of the CPI task on the DAVIS dataset. The evaluation was performed across 1000 molecules generated the models; Table S3: Experimental results of the CPI task on the DAVIS dataset. Figure S1: Comparison of the QED (Quantitative Estimate of Drug-likeness), SAS (Synthetic Accessibility Score), and LogP (Octanol-Water Partition Coefficient) distributions for molecules in the JAK2 train set and those generated by two variants of OMG-GPT.
