# Supplementary material for: Enhancing Unconditional Molecule Generation via Online Knowledge Distillation of Scaffolds
Source: Molecules. 2025 Mar 12;30(6):1262. doi: 10.3390/molecules30061262 (PMC11945111; doi:10.3390/molecules30061262)
Supplement: Supplementary file 1 [file molecules-30-01262-s001.zip › molecules-3434996-supplementary.pdf]

Supplementary Material:
Enhancing Unconditional Molecule Generation via Online Knowledge Distillation of Scaffolds

Huibin Wang[1], Zehui Wang[1], Minghua Shi[1], Zixian Cheng[1], Ying Qian[1]

（1. Shanghai Frontiers Science Center of Molecule Intelligent Syntheses, School of Computer Science and Technology, East China Normal University, 3663 North Zhongshan Road, Putuo District, Shanghai, 200062, Shanghai, China）

A Additional Experiments

A.1 Effects of Different Loss Weight Settings for Unconditional Generation

We examined the effect of different loss function weights on generation performance}, varying the values λ_1, λ_2 and λ_3 in equation (5) of the main text. The weight values are selected from {0.5, 1, 1.5} to explore different configurations. The results of these experiments are summarized in Table S1.

Table S1. Comparison of OMG's performance with different weights of losses on the MOSES dataset.

**Weight Configuration**			**Evaluation Metrics**			
$\boldsymbol{\lambda_1}$	$\boldsymbol{\lambda_2}$	$\boldsymbol{\lambda_3}$	**Validity**	**Uniqueness**	**Novelty**	$\boldsymbol{IntDiv_1}$
1	1	1	0.980	**1.0**	0.936	0.855
1	1.5	0.5	**0.990**	**1.0**	0.895	0.854
1	0.5	1.5	0.962	**1.0**	**0.958**	0.855
1.5	1	0.5	**0.990**	**1.0**	0.903	0.855
0.5	1	1.5	0.960	**1.0**	0.957	**0.856**
1.5	0.5	1	0.981	**1.0**	0.935	0.855
0.5	1.5	1	0.982	**1.0**	0.938	0.855

The best results is in **bold.**

We used the results with $\lambda_1 = 1, \lambda_2 = 1, \lambda_3 = 1$ as a reference. When λ_1 is fixed at 1, increasing λ_2 while decreasing λ_3 improved validity by 1.1% but reduced novelty by 4.1%. Conversely, decreasing λ_2 while increasing λ_3 lowers validity by 1.8% but raises novelty by 2.2%. Similarly, when λ_2 is fixed at 1, the validity and the novelty exhibit the same trend as observed in the previous case, varying inversely with the adjustments of weights.

However, when λ_2 is fixed at 1, variations in λ_1 and λ_2 result in minimal changes (within 0.1%) across all metrics. Additionally, the uniqueness remained stable regardless of weight adjustments.

These results indicate that the molecular generation losses associated with λ_1 and λ_2 play the most significant role in ensuring the validity of the generated molecules. Meanwhile, the mutual learning loss controlled by λ_3 has the greatest impact on enhancing novelty. Among the tested configurations, we found that setting $\lambda_1 = 1.5, \lambda_2 = 1, \lambda_3 = 0.5$ achieves a well-balanced

performance across all metrics. Therefore, this configuration was selected for conducting the final experiments.

A.2 Transfer Learning Experiments

We set up two downstream tasks to test the generalizability of the OMG training framework in more scenarios related to molecular structure learning. The first task involves unconditionally generating specific molecules that adhere to specific property patterns, while the second task focuses on a classification task that determines whether a given molecule would interact with specific proteins.

A.2.1 Additional Evaluation Metrics

For the unconditional generation task, we use the same metrics as mentioned in the main text, including validity, unique rate, and novelty, etc. For the classification task, we introduce three commonly used evaluation metrics: Precision, Recall, and F1 score.

Precision Precision measures the proportion of true positive predictions among all samples that were predicted as positive by the model. It is formally defined as:

$$\text{Precision} = \frac{TP}{TP+FP}.$$

Where the term TP represents the number of instances which are predicted as positive and FP represents the number of instances predicted as negative.

Recall measures the proportion of correctly predicted positive samples out of all truly positive samples, which is calculated as:

$$\text{Recall} = \frac{TP}{TP+FN}.$$

Where the term FN represents the number of instances which are predicted as negatives.

F1 Score is the harmonic mean of precision and recall, providing a comprehensive evaluation of the model's ability to balance precision and recall. It is calculated as follows:

$$\text{Precision} = 2 \cdot \frac{\text{Precision}\cdot\text{Recall}}{\text{Precision}+\text{Recall}}.$$

A.2.1.1 Unconditional Generation of Specific Molecules

To evaluate OMG's application in the unconditional generation of a specific molecule set that adheres to specific property patterns, we adopted the dataset used in [49]. This dataset collects molecules derived from ChEMBL that have been experimentally validated as inhibitors for the specific protein JAK2. For simplicity, the adopted dataset is named JAK2.

Considering that the molecular SMILES string lengths and property values in the Guacamol dataset exhibit greater variability than the MOSES dataset, models trained on this dataset may be able to uncover more complex associations between molecular structures and potential properties. Therefore, we selected the OMG-GPT and OMG-SG models trained on the Guacamol dataset and fine-tuned them on JAK2. To increase the difference in data distribution, we filtered out SMILES strings in the JAK2 dataset that overlap with those in the Guacamol dataset, resulting in 1,772 SMILES strings used for fine-tuning. The fine-tuning process was still based on the OMG training

framework, using the same parameters described in the manuscript. During testing, the fine-tuned OMG-GPT generated 1,000 SMILES strings for evaluation. In addition to evaluating the validity, unique rate, and novelty, we also assessed the distribution of three property values (QED, SAS, LogP). Furthermore, we used GraphDTA [40] to evaluate the distribution of pIC50 values for these molecules on the JAK2 protein. For comparison, the OMG-GPT trained on Guacamol and the OMG-GPT model trained from scratch on JAK2 based on the OMG framework were selected. The experimental results are shown in Table S2 and Figure S2.

Table S2. Experimental results of the CPI task on the DAVIS dataset. The evaluation was performed across 1000 molecules generated the models.

**Model**	**Validity**	**Unique**	**Novelty**	**pIC50 (Mean/Std.) JAK2** (7.13/0.71)
OMG-GPT (Guacamol)	0.858	**1.0**	0.99	5.57/0.51
OMG-GPT (JAK2)	0.004	**1.0**	**1.0**	**-**
OMG-GPT (Guacamol+JAK2)	**0.903**	0.92	**1.0**	6.83/0.64

- **JAK2 (**7.13/0.71**)** means that the mean value and standard deviation value of pIC50 scores for molecules in the selected dataset against the protein JAK2 are 7.13 and 0.71, respectively.
- The suffixes (Guacamol) and (JAK2) denote the OMG-GPT was solely trained in the Guacamol dataset or the JAK2 dataset respectively. The suffix (Guacamol + JAK2) denotes the OMG-GPT was pretrained on the Guacamol dataset and fine-tuned on the JAK2 dataset.
- The best result for the metrics including validity, unique rate and novelty is in **bold**.

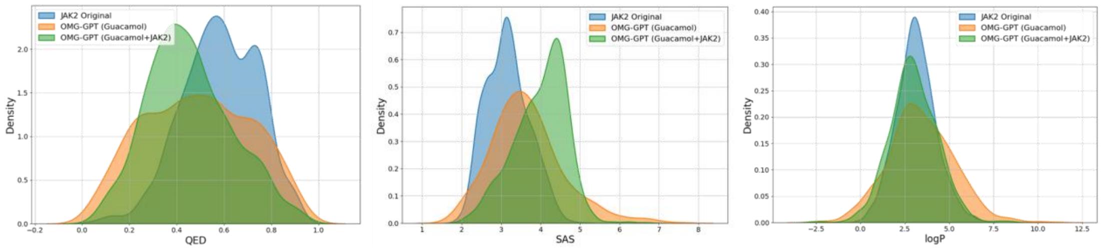

Figure S1. Comparison of the QED (Quantitative Estimate of Drug-likeness), SAS (Synthetic Accessibility Score), and LogP (Octanol-Water Partition Coefficient) distributions for molecules in the JAK2 train set and those generated by two variants of OMG-GPT .

OMG-GPT (JAK2), which was trained from scratch on the JAK2 dataset, only produced 4 valid SMILES strings among the 1000 generated samples (the property-specific evaluation on the generated molecules was thus ignored), indicating a challenging task in identifying valid molecular structures within the JAK2 dataset. OMG-GPT (Guacamol) generated 858 valid SMILES, whereas OMG-GPT (Guacamol+JAK2) produced 903 valid SMILES, improving both validity and novelty. These results indicate that the model, pre-trained on the Guacamol dataset, retained its ability to generate valid and novel molecules while also benefiting from the additional valuable molecular structural information learned from the JAK2 dataset.

In terms of the molecular properties, the mean value of pIC50 scores for the generation molecules of OMG-GPT (Guacamol+JAK2) is closer to the mean pIC50 score of original molecules in the JAK2 dataset, compared to the molecules generated by OMG-GPT (Guacamol).

This suggests that the fine-tuning process allows the model to effectively transfer the knowledge associated with molecular properties to the downstream domain. Furthermore, from the KDE distribution plots of QED, SAS, and LogP, the distribution shapes of the generated results from OMG-GPT (Guacamol+JAK2) closely resemble the distribution of the JAK2 dataset. However, there are still noticeable differences in the value range compared to the JAK2 dataset. Additionally, the three molecular sets exhibit a significant overlap in the property value ranges. This further indicates that the knowledge related to the association between molecular structures and properties in the Guacamol dataset has largely been preserved, and the knowledge also includes certain invariant content, demonstrating the generalizability of our approach.

A.2.1.2 Compound-Protein-Interaction Prediction

Given the power of OMG of learning molecular structures, we also assess the OMG framework in the application of Compound-Protein-Interaction (CPI) prediction task which is closely associated to the molecular structures. The CPI task can be framed as a binary classification problem, where the goal is to predict whether a given pair of proteins and compounds interact.

We implemented UniT [50] for the task as a reference, which consists of two transformer encoders incorporating a protein sequence and SMILES string of a compound molecule, respectively. The configuration of the transformer encoder and OMG-GPT, except for the MLP used for online distillation, is identical, consisting of 8 encoder layers. Each layer features an 8-head attention mechanism, and the dimensionality of the features is set to 256. During fine-tuning, we injected parameters of the OMG-GPT which was pretrained in the Guacamol dataset for unconditional molecule generation task into the transformer encoder of the compound part, referred to as UniT (OMG-GPT).

The comparison of the performance between the UniT and UniT (OMG-GPT). We used the public DAVIS dataset [51] to train (fine-tune) and evaluate the prediction models, with F1, accuracy and recall as evaluation metrics. The evaluation results were collected in Table S3.

Table S3. Experimental results of the CPI task on the DAVIS dataset.

**Models**	**Precision**	**Recall**	**F1**
UniT	0.844	0.837	0.841
UniT (OMG-GPT)	**0.848**	**0.847**	**0.845**

The suffix (OMG-GPT) means the model uses the parameters of the OMG-GPT pretrained on the Guacamol dataset. The best results is in **bold.**

Compared to UniT trained from scratch, UniT (OMG-GPT) demonstrated slight but overall improvements. This indicates that even though the learning task during the pretraining phase of OMG-GPT differs from the downstream task, it is still able to learn useful structural knowledge about molecules from the data. This knowledge can, to some extent, assist with tasks related to molecular structures. This perspective also suggests that the model trained under the proposed OMG framework has a certain degree of generalizability.
